# Induction of apoptosis by ethanol extract of sapodilla leaf in HGT-1 cells involved caspase-independent signal transduction pathway and activation of Nrf2/HO-1

**DOI:** 10.3389/fphar.2026.1803513

**Published:** 2026-03-03

**Authors:** Bee Ling Tan, Lee Chin Chan

**Affiliations:** 1 Department of Diagnostic and Allied Health Science, Faculty of Health and Life Sciences, Management and Science University (MSU), University Drive, Shah Alam, Selangor, Malaysia; 2 Biovalence Sdn. Bhd., Taman Mayang, Petaling Jaya, Selangor, Malaysia

**Keywords:** apoptosis, Bcl-2, caspase-8, cytotoxicity, gastric cancer

## Abstract

Gastric cancer has become one of the most common cancers globally. Sapodilla or known as *Manilkara zapota* (L.) P. Royen, has been traditionally applied for cough, cold, and diarrhea. The detailed mechanisms in modulating sapodilla leaf 70% ethanol extract against HGT-1 human gastric cancer cell line have yet to be fully studied. Apoptosis induction was evaluated using an Annexin V-FITC/PI staining kit with flow cytometry. Caspase-3 and -8 expression were determined by colorimetric analysis. To explore the molecular mechanisms of action of the 70% ethanol extract of sapodilla leaves in HGT-1 cells, quantitative real-time PCR (qPCR) was performed. Overall analyses demonstrated that sapodilla leaf 70% ethanol extract was cytotoxic and suppressed the growth of HGT-1 cells. The 70% ethanol extract of sapodilla leaves significantly suppressed the proliferation of HGT-1 cells. Moreover, it enhanced the expression of nuclear factor E2-related factor 2 (*Nrf2*) and heme oxygenase-1 (*HO-1*), while concurrently suppressing the transcriptional activities of nuclear factor-kappa B (*NF-κB*) and inducible nitric oxide synthase (*iNOS*). Results indicate that sapodilla leaf 70% ethanol extract has an anti-gastric cancer effect via modulation of multiple signaling pathways. Sapodilla leaf 70% ethanol extract demonstrates promising potential as an anti-gastric cancer agent.

## 1 Introduction

Gastric cancer has become the sixth primary malignancy globally (World Health Organization, 2019). An estimated 1.1 million new cases of gastric cancer and 770,000 related deaths occurred in 2020 worldwide, with incidence rates approximately twice as high in males compared to females (International Agency for Research on Cancer, 2026). The yearly gastric cancer-related health burden is expected to increase to nearly 1.3 million deaths and 1.8 million new cases by 2040, accounting for 66% and 63%, respectively, compared to 2020 (International Agency for Research on Cancer, 2026). Despite many efforts that have been made to improve the available therapeutic approaches, evidence from various studies suggests that, even in economically advanced countries, survival outcomes for gastric cancer remain suboptimal (Morgan et al., 2022). Therefore, the potential of natural products in yielding new anticancer drugs has become a focal point in recent scientific investigations.

Due to its key regulatory role in gastric cancer progression, NF-κB is regarded as a molecular target with potential for therapeutic intervention (Chaithongyot et al., 2021). Emerging evidence has demonstrated that herbal medicine downregulates lipopolysaccharide-induced iNOS expression via NF-κB inactivation (Lei et al., 2016). Apart from NF-κB activity, Nrf2 stimulates phase II enzyme through interaction with antioxidant responsive element (ARE) sequences (Ulasov et al., 2022). Compelling evidence has revealed that induction of Nrf2 pathway could ameliorate oxidative stress in cancer through modulation of phase II antioxidant enzymes, for instance, heme oxygenase-1 (HO-1) (Wang et al., 2022).

The predominant etiological factors involved in gastric cancer are *Helicobacter pylori* infection and diet (Kumar et al., 2021; Maddineni et al., 2022). Frequent consumption of foods preserved through salting, smoking, or pickling is considered to be associated with a higher risk of developing gastric cancer (Yoo et al., 2020). Dietary patterns that emphasize high intake of vegetables and fruits have been shown a protective effect against gastric cancer (Dalmartello et al., 2022; Kermanshahi et al., 2023). Over the past few decades, extensive studies have shown an association between natural compounds and gastric carcinogenesis (Vitelli-Storelli et al., 2020; Gunes-Bayir et al., 2022). Traditional medicinal plants exert antioxidant activity and have shown an ability to suppress cancer cells (Tan et al., 2018a; Tan et al., 2018b; Tan et al., 2023; Jamieson et al., 2023; Tan et al., 2025). Thus, it is suggested that the traditional plants may have preventive effects against tumor initiation and progression and thus may offer therapeutic benefits.

Sapodilla (*Manilkara zapota* (L.) P. Royen) is a tropical evergreen tree native to Mexico and Central America, now widely cultivated in the Indian subcontinent (Ghani, 2003). Folk medicine practices have utilized the leaf for managing conditions including cough, cold, diarrhea, and pulmonary infections (Kaur et al., 2020). Our earlier research showed that methanolic extract of *Manilkara zapota* leaves effectively inhibited the proliferation of HeLa cervical cancer cells (Tan et al., 2018a). Nevertheless, the chemopreventive activity of 70% ethanolic sapodilla leaf extract against gastric cancer, particularly in HGT-1 cells, remains poorly understood. Accordingly, this study sought to investigate the underlying mechanisms involved in its activity.

## 2 Materials and methods

### 2.1 Materials and cell culture

A panel of cell lines comprising mouse fibroblast (BALB/c 3T3), human prostate carcinoma (PC-3), cervical cancer (HeLa), gastric carcinoma (HGT-1), colorectal adenocarcinoma (HT-29), colon carcinoma (HCT-116), and hepatocellular carcinoma (HepG2) was utilized in this study, all of which were sourced from the American Type Culture Collection (ATCC). 0.25% trypsin-EDTA, RPMI-1640 and DMEM media, 100× penicillin-streptomycin solution, and MycoPLEx™ fetal bovine serum, were sourced from Gibco (Grand Island, NY, USA). Quantification of apoptosis-regulating proteins Bcl-2 and Bax was performed using Human SimpleStep ELISA® kits from Abcam (Cambridge, United Kingdom). All additional chemicals utilized in the experiments were of analytical grade and purchased from Sigma-Aldrich (St. Louis, MO, USA).

### 2.2 Sample collection

Sapodilla leaf was acquired from Pahang, Malaysia, and taxonomically authenticated by Dr. Mohd Firdaus Ismail at the Institute of Bioscience, Universiti Putra Malaysia. The voucher specimen was archived under reference number SK 3179/17.

### 2.3 Plant extraction

Following washing and chopping, sapodilla leaves were extracted using a slightly modified method based on previous literature (Tan et al., 2018a). Five grams of leaf material were soaked in 40 mL of 70% ethanol and incubated at 40 °C with continuous stirring for 2 h. The mixture was then filtered, and the solvent was removed at 40 °C using a rotary evaporator to obtain the crude extract.

### 2.4 Cell lines

The HGT-1, HCT-116, and HT-29 cell lines were maintained in DMEM enriched with 100 μg/mL streptomycin, 100 IU/mL penicillin, and 10% (v/v) FBS. In contrast, HepG2, BALB/c 3T3, PC-3, and HeLa cell lines were cultured in RPMI-1640 medium with the same supplements. All cultured cells were incubated at 37 °C in a humidified environment with 5% CO_2_.

### 2.5 Evaluation of cytotoxicity of sapodilla leaf 70% ethanol extract

The cytotoxicity of the 70% ethanol extract of sapodilla leaf was evaluated in both normal (BALB/c 3T3) and cancerous cell lines (PC-3, HepG2, HeLa, HGT-1, HCT-116, and HT-29) using MTT and LDH assays. For the assay, approximately 5 × 10^4^ cells were seeded per well in a 96-well plate and incubated for 24 h to promote attachment. Cell was subsequently treated with various doses of the extract, ranging from 1.563 to 200 μg/mL. Following treatment, 20 μL of 5 mg/mL MTT solution was added to each well and incubated for 4 h. Thereafter, 100 μL of dimethyl sulfoxide was introduced to solubilize the formazan crystals. The absorbance was then recorded at 570 nm, with a reference wavelength of 630 nm. Untreated cells, including both cancer lines and BALB/c 3T3 fibroblasts, served as negative controls.

### 2.6 Lactate dehydrogenase assay

Cytotoxicity assessment was carried out by *in vitro* Toxicology Assay Kit in accordance with manufacturer’s instructions. BALB/c 3T3 fibroblasts and cancer cells (PC-3, HepG2, HeLa, HGT-1, HCT-116, and HT-29) were plated at 5 × 10^4^ cells per well in a 96-well plate and incubated for 24 h to promote attachment. Subsequently, cancer cell lines were exposed to different concentrations (1.563–200 μg/mL) of sapodilla leaf 70% ethanol extract for 72, 48, and 24 h. Untreated cells from both cancer and normal cell lines served as controls. Lactate dehydrogenase released into the culture medium was quantified by measuring absorbance at 490 nm to assess cell membrane integrity and cytotoxicity.

### 2.7 Cell morphological of sapodilla leaf 70% ethanol extract-treated cells

HGT-1 cells were seeded into 6-well plates at a density of 1 × 10^5^ cells per well and incubated for 24 h to allow attachment. The cells were then treated with sapodilla leaf extract prepared in 70% ethanol at doses of 84, 42, and 21 μg/mL for 72 h. Following treatment, cellular morphological alterations associated with apoptosis or necrosis were examined using an inverted light microscope (Olympus, PA, United States), and representative images were acquired for further analysis.

### 2.8 Cell cycle analysis

Cell cycle distribution was analyzed using the CycleTEST™ PLUS DNA Reagent Kit according to the manufacturer’s instructions. HGT-1 cell line was treated with sapodilla leaf extract prepared in 70% ethanol for 72 h. After treatment, cells were collected by centrifugation and resulting pellet was resuspended in the buffer. Subsequently, a total of 250 μL trypsin buffer and 200 μL trypsin inhibitor/RNase buffer were added, followed by a 10-min incubation. Propidium iodide (200 μL) was then added for DNA staining. Flow cytometry was performed with a NovoCyte flow cytometer (ACEA Biosciences, Inc.) to evaluate cell cycle phase distribution.

### 2.9 Determination of apoptotic cell death

Apoptotic profiling was conducted using the Annexin V-FITC Apoptosis Detection Kit I, based on the protocol provided by the manufacturer. HGT-1 cell line was plated seeded at a density of 1 × 10^5^ cells in 25 cm^2^ culture flasks and incubated for 24 h to allow attachment. The cell was then treated with sapodilla 70% ethanol leaf extract at doses of 84, 42, and 21 μg/mL for 72 h. Following treatment, cells were trypsinized and subsequently washed twice with EDTA-BSA-PBS buffer. The cell pellet was gently resuspended in 100 μL of 1× binding buffer, followed by staining with 5 μL Annexin V-FITC and 10 μL propidium iodide. After a 10-min incubation in the dark, the sample was diluted with an additional 400 μL of 1× binding buffer. Apoptotic cell populations were subsequently evaluated using a FACSCalibur flow cytometer (BD Biosciences, United States).

### 2.10 Quantification of Bcl-2 and bax protein

The expression of Bcl-2 and Bax protein was quantified using Human SimpleStep ELISA® Kits, based on the protocol provided by the manufacturer. HGT-1 cell line was plated at a density of 1 × 10^5^ cells in 25 cm^2^ culture flasks and incubated for 24 h to allow attachment. Cell was then exposed to sapodilla 70% ethanol leaf extract at doses of 84, 42, and 21 μg/mL for 72 h. After treatment, cells were collected and spun at 500 × *g* for 5 min at 4 °C, after which the supernatant was retrieved for protein quantification. Equal volumes (50 μL) of sample or standard were added to 50 μL antibody cocktail per well of ELISA plate and incubated for 1 h at room temperature. Next, 100 μL of TMB substrate was added, followed by centrifugation at 400 × *g* for 10 min. The reaction was stopped with 100 μL of stop solution, and absorbance was measured at 450 nm.

### 2.11 Caspase-8 and caspase-3 analysis

Caspase activity was evaluated using commercially available colorimetric assay kit, in accordance with the manufacturer’s instructions. HGT-1 cell line was plated into 25 cm^2^ flasks at a density of 1 × 10^5^ cells for 24 h. The cell line was then exposed to sapodilla leaf extract (70% ethanol) at doses of 84, 42, and 21 μg/mL for 72 h. After cell lysis, 25 μL of pre-chilled lysis buffer was added to each sample. To evaluate caspase activity, 50 μL of either 2× Reaction Buffer 8 (for caspase-8) or 2× Reaction Buffer 3 (for caspase-3) was mixed with lysates, along with 5 μL of the respective colorimetric substrate (DEVD-pNa for caspase-3 or IETD-pNa for caspase-8). The sample was incubated at 37 °C for 2 h, followed by absorbance measurement at 405 nm using a microplate reader.

### 2.12 Determination of quantitative real-time polymerase Chain reaction (PCR)

Total RNA was extracted using TRI Reagent® according to the manufacturer’s protocols. cDNA was synthesized from 2 μg of total RNA in a 20 μL reaction volume using an Authorized Thermal Cycler (Eppendorf, NY, United States). Quantitative real-time PCR (qRT-PCR) was conducted on the CFX platform using SYBR® Select Master Mix, with each reaction including both samples and controls (run in triplicate). Amplification and fluorescence detection were carried out using the BioRAD-iQ™ 5 Multicolor Real-Time PCR Detection System (Hercules, CA, United States), and the resulting data were evaluated by CFX Manager™ software (Table 1).

**TABLE 1:** The nucleotide primer sequences derived from human cell lines and sourced from the GenBank database.

Name of primer (accession number)	Oligonucleotides (5′-3′) sequence
*Nrf2* [BC011558.1]	F-GCGACGGAAAGAGTATGAGC R-TGGGAGTAGTTGGCAGATCC
*HO-1* [NM_002133.2]	F-CTTCTTCACCTTCCCCAACA R-GCTCTGGTCCTTGGTGTCAT
*NF-κB* ^b^ [M58603]	F-TGGAAGCACGAATGACAGAG R-TGAGGTCCATCTCCTTGGTC
*iNOS* [AF049656.1]	F-GTGGTGACAAGCACATTTGG R-GTCATGAGCAAAGGCACAGA
*ACTB* ^a^ ^,^ ^b^ [NM 001101.3]	F-AGAGCTACGAGCTGCCTGAC R-AGCACTGTGTTGGCGTACAG
*GAPDH* ^a^ ^,^ ^b^ [NM 002046.4]	F-GGATTTGGTCGTATTGGGC R-TGGAAGATGGTGATGGGATT
18S rRNA^a^ ^,^ ^b^ [HQ387008.1]	F-GTAACCCGTTGAACCCCATT R-CCATCCAATCGGTAGTAGCG

*Nrf2*: nuclear factor E2-related factor 2; NF-κB: nuclear factor-kappa B; *GAPDH*: glyceraldehyde-3-phosphate dehydrogenase; *ACTB*: beta-actin; *HO-1*: heme oxygenase-1; *iNOS*: inducible nitric oxide synthase.

^a^ Housekeeping gene.

^b^ Tan et al. (2018a).

### 2.13 Qualitative phytochemical screening of sapodilla leaf 70% ethanol extract

The qualitative phytochemical analysis of sapodilla leaf 70% ethanol extract was conducted for the presence of triterpenoids, phlobatannins, saponins, steroids, and flavonoids according the procedures as described earlier by Tan et al. (2018a).

### 2.14 Determination of total phenolic content

The Folin–Ciocalteu colorimetric assay was employed to assess the total phenolic content of the sapodilla leaf extracts, with slight modifications based on the procedure described by Singleton and Rossi (1965). Briefly, 0.3 mL of the extracts were combined with 1.5 mL of Folin–Ciocalteu reagent, followed by the addition of 1.2 mL of sodium carbonate (7.5% w/v). The resulting solution was vortexed thoroughly and left to incubate at ambient conditions for 30 min. The absorbance was then recorded at 765 nm using a UV-visible spectrophotometer, and total phenolic content expressed as milligrams gallic acid equivalents per gram of extract (mg GAE/g extract).

### 2.15 Total flavonoid content quantification

The colorimetric method was used to quantify total flavonoids in the sapodilla leaf extract (Shanmugapriya et al., 2011). In brief, 0.5 mL of the extract was added into 1.5 mL of 95% ethanol, then 0.1 mL of 1 M potassium acetate and 0.1 mL of 10% aluminum chloride hexahydrate were added. The reaction volumes were brought to 5.0 mL by adding 2.8 mL of distilled water. The solutions were incubated at ambient conditions for 40 min, and the absorbance was subsequently read at 415 nm using a UV-visible spectrophotometer. Total flavonoid content was determined and reported as milligrams of quercetin equivalent per 100 g of extract (mg QE/100 g extract).

### 2.16 1, 1-diphenyl-2-picryl-hydrazyl (DPPH) radical scavenging capacity analysis

DPPH radical scavenging activity of the sapodilla leaf extracts were assessed using a UV-Visible spectrophotometric method as previously described (Zhang and Hamauzu, 2004), with slight modifications. Briefly, 0.5 mL of the plant extract was added into 1.5 mL of 0.1 mM DPPH solution prepared in methanol. The solution was vortexed for 15 s and then allowed to incubate in the dark at ambient condition for 1 h. A spectrophotometric reading was determined at 517 nm. Antioxidant activity was expressed as the effective concentration required to scavenge 50% of DPPH radicals (EC_50_).

### 2.17 Beta-carotene bleaching test analysis

Beta-carotene bleaching assay was conducted following a modified method by Tan et al. (2013). About 5 mg of β-carotene was dissolved in 10 mL of chloroform. Vacuum evaporation at 40 °C was employed to eliminate chloroform. An emulsion was formulated by combining the resulting residue with 100 mL of distilled water, 400 mg of Tween 40, and 40 mg of linoleic acid. The prepared emulsions were distributed into test tubes, and the initial absorbance (time zero) was recorded at 470 nm. The tubes were incubated at 50 °C, and absorbance readings were taken every 20 min for a total duration of 2 h to monitor the rate of β-carotene degradation.

### 2.18 Quantification of polyphenolic compounds using UPLC

Polyphenolic compounds were quantified using an Agilent 1290 Infinity HPLC system (Model G4220A) attached with a diode array detector (DAD). Detection was carried out at wavelengths of 320 nm and 280 nm. Separation of components was achieved via chromatography using a LiChroCART® 250–4, 6 C18 column (250 mm × 4.6 mm, 5 μm particle size). A mobile phase employed was a mixture of solvent B (acetonitrile) and solvent A (water:acetic acid, 94:6, v/v; pH 2.27). A gradient elution was employed as follows: 0%–15% B over 40 min, 15%–45% B over the next 40 min, and 45%–100% B over the final 10 min, at a constant flow rate of 0.5 mL/min (Chirinos et al., 2009). Identification and quantification were performed using external standards, including vanillic acid, ferulic acid, caffeic acid, gallic acid, syringic acid, and p-coumaric acid.

### 2.19 Statistical analysis

Data were analyzed using SPSS version 28.0 and expressed as mean ± standard deviation. One-way ANOVA followed by Tukey’s *post hoc* test was used for multiple group comparisons. Differences were considered statistically significant at *P* < 0.05.

## 3 Results and discussion

### 3.1 Suppression of cell viability by sapodilla leaf 70% ethanol extract

The antiproliferative effects of sapodilla leaf 70% ethanol extract on cancer cells, PC-3, HepG2, HeLa, HGT-1, HCT-116, and HT-29 along with BALB/c 3T3 cells were examined by exposing them to different doses of extract for 72, 48, and 24 h using 3-(4,5-dimethylthiazol-2-yl)-2,5-diphenyltetrazolium bromide (MTT) assay. Exposure of HGT-1 cells to sapodilla leaf 70% ethanol extract (1.563–200 μg/mL) led to a time-dependent reduction in cell proliferation (Table 2). Our data showed that sapodilla leaf 70% ethanol extract had the greatest antiproliferative activity against the HGT-1 cells after 24, 48, and 72 h as opposed to the other cancer cells analyzed, with 156.98 ± 8.98, 97.78 ± 6.45, and 41.98 ± 5.68 μg/mL, respectively. Additionally, sapodilla leaf 70% ethanol extract also suppressed the proliferation of HT-29 cell lines, with IC_50_ values 101.16 ± 16.33 μg/mL (24 h), 99.93 ± 7.78 μg/mL (48 h), and 62.03 ± 4.59 μg/mL (72 h). Similarly, sapodilla leaf 70% ethanol extract also suppressed the growth of HCT-116 cells after 24 h (109.23 ± 9.22 μg/mL), 48 h (98.31 ± 5.22 μg/mL), and 72 h (74.97 ± 5.32 μg/mL). Among the colon cancer cells (HCT-116 and HT-29), HT-29 (62.03 ± 4.59 μg/mL) were more sensitive towards sapodilla leaf 70% ethanol extract than HCT-116 (74.97 ± 5.32 μg/mL) after 72 h incubation. Likewise, sapodilla leaf 70% ethanol extract reduces the growth of HepG2 and HeLa cell lines. Of all the cancer cell lines studied, sapodilla leaf 70% ethanol extract showed less potent antiproliferative activity against HepG2 cells (102.85 ± 6.95 μg/mL) (Table 2). 70% ethanol was used for extraction in this study because the solvent penetration ability is strong, which can extract non-polar and polar compounds in one extraction. Indeed, these extraction methods do not favor the extraction of any specific bioactive constituents (Liu, 2008).

**TABLE 2:** Treatment of sapodilla leaf 70% ethanol extract (1.563-200 μg/mL) on several cancer cells for 72, 48, and 24 h measured by LDH and MTT techniques.

Cancer cells	MTT (μg/mL)			LDH (μg/mL)		
	24 h	48 h	72 h	24 h	48 h	72 h
HT-29	101.16 ± 16.33^a^	99.93 ± 7.78^a^	62.03 ± 4.59^b^	154.67 ± 3.32^a^	119.18 ± 4.96^b^	96.03 ± 14.09^c^
HCT-116	109.23 ± 9.22^a^	98.31 ± 5.22^a^	74.97 ± 5.32^b^	103.22 ± 9.22^a^	85.21 ± 11.20^ab^	77.92 ± 5.97^b^
HeLa	790.43 ± 12.56^a^	179.02 ± 5.34^b^	40.93 ± 2.19^c^	767.32 ± 15.97^a^	153.22 ± 8.41^b^	63.15 ± 9.55^c^
HGT-1	156.98 ± 8.98^a^	97.78 ± 6.45^b^	41.98 ± 5.68^c^	123.07 ± 9.34^a^	95.64 ± 5.49^b^	47.65 ± 7.56^c^
HepG2	141.32 ± 12.76^a^	115.82 ± 9.35^a^	102.85 ± 6.95^a^	125.67 ± 10.21^a^	123.65 ± 9.03^a^	98.23 ± 7.66^b^

HCT-116: human colon carcinoma; HeLa: human cervical cancer; HepG2: human hepatocellular carcinoma; HGT-1: human gastric adenocarcinoma; HT-29: human colorectal adenocarcinoma; LDH: lactate dehydrogenase; MTT: 3-(4,5-dimethylthiazol-2-yl)-2,5-diphenyltetrazolium bromide.

All data are presented as mean ± SD (n = 3). ^a, b, c^ Values with different superscript letter in the same row for their respective assays are significantly different according to Tukey’s test (*P* < 0.05).

**FIGURE 1:**
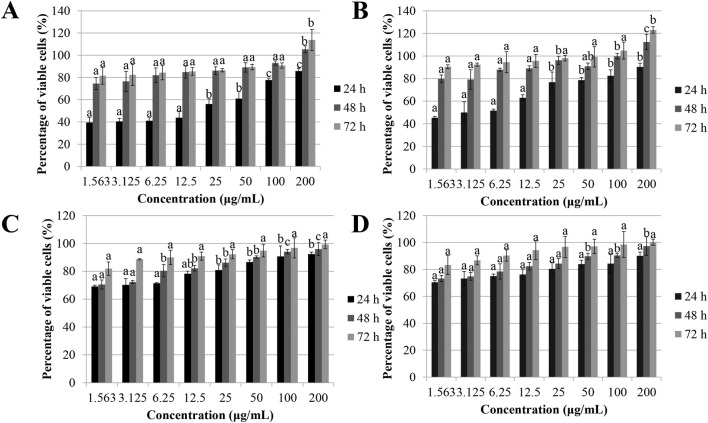
Effect of sapodilla leaf 70% ethanol extract on cancer and non-cancerous cell lines. **(A)** The cell viability after 24, 48, and 72 h exposure with sapodilla leaf 70% ethanol extract on human prostate cancer (PC-3) cell line using MTT assay. **(B)** sapodilla leaf 70% ethanol extract increases the viability of PC-3 cell line after 24, 48, and 72 h evaluated using LDH assay. **(C)** The cell viability of mouse fibroblast (BALB/c 3T3) cell line evaluated by MTT assay. **(D)** Treatment of sapodilla leaf 70% ethanol extract in BALB/c 3T3 cells using LDH assay. The data are presented as mean ± SD (n = 3). The data with different superscript letter indicates a significant difference between groups by Tukey’s test (*P* < 0.05).

Figure 1A illustrates the percentage of viable PC-3 cell line after exposure to sapodilla leaf 70% ethanol extract for 24, 48, and 72 h. We found that incubation with sapodilla leaf 70% ethanol extract increased the PC-3 cells viability. Among the cancer cells tested, PC-3 cells exhibited relatively higher resistance to the sapodilla leaf extract. Nonetheless, further study is warranted to investigate the mode of action in sapodilla leaf 70% ethanol extract-treated cells.

To validate the antiproliferative effect of the 70% ethanol extract of sapodilla leaf on cancer cells, the proliferation of all cancer cells studied were evaluated by LDH assay. Following treatment with the extract (1.563–200 μg/mL), LDH technique was conducted to determine cell membrane integrity. As shown in Table 2, extract treatment resulted in time-dependent reduction of cell viability in HCT-116, HT-29, and HeLa cells. Sapodilla leaf 70% ethanol extract was shown relatively potent inhibitory effects on the HGT-1 cell lines, with an IC_50_ value of 47.65 ± 7.56 μg/mL at 72 h. However, the findings indicated that HepG2 cells exhibited lower sensitivity to the extract in comparison to HGT-1 cells. Likewise, an increase in PC-3 cell viability was observed at all tested time points (24, 48, and 72 h) based on LDH measurements following extract treatment (Figure 1B). Notably, no cytotoxic was found in BALB/c 3T3 cells after incubation with sapodilla leaf 70% ethanol extract as determined by LDH and MTT assays (Figures 1C,D). Collectively, our findings showed that sapodilla leaf 70% ethanol extract can promote cytotoxicity in cancer cells studied, in which sapodilla leaf 70% ethanol extract showed the most suppressive effects on HGT-1 cells. Given the wide range of cytotoxic effects observed in HGT-1 cell line treated with sapodilla leaf 70% ethanol extract, as evidenced by both MTT and LDH assays, the doses (84, 42, and 21 μg/mL) and HGT-1 cells were selected for further analysis. From the data obtained, sapodilla leaf 70% ethanol extract reduced cell proliferation after treatment for 72 h. Thus, a 72-h incubation period was chosen for subsequent analyses.

### 3.2 Sapodilla leaf 70% ethanol extract triggers changes of morphology in HGT-1 cells

**FIGURE 2:**
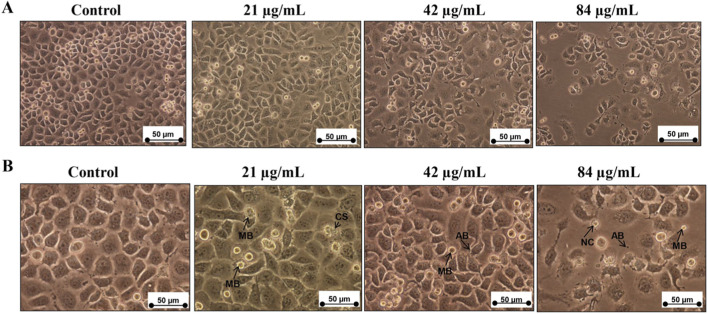
Changes of morphology in HGT-1 cells incubated with 21, 42, and 84 μg/mL of sapodilla leaf 70% ethanol extract for 72 h, and observed under an inverted light microscope. **(A)** Sapodilla leaf 70% ethanol extract triggers changes in morphology and reduces the growth of HGT-1 cells (Magnification ×200). **(B)** Close-up view of changes in morphology of HGT-1 cells after exposure to sapodilla leaf 70% ethanol extract (Magnification ×400). The HGT-1 cells demonstrated the typical characteristics of apoptosis, for instance, membrane blebbing (MB), apoptotic bodies (AB), cellular shrinkage (CS), and nuclear compaction (NC).

Our data showed that increasing the concentration of sapodilla leaf 70% ethanol extract for 72 h from 21 to 84 μg/mL induced morphological changes and decreased the viability of HGT-1 cells (Figure 2A). HGT-1 cells showed typical morphological changes upon exposure to various doses of the extract for 72 h (Figure 2B). We observed that HGT-1 cells began to change the appearance at 21 μg/mL of sapodilla leaf 70% ethanol extract. Treatment with sapodilla leaf 70% ethanol extract in HGT-1 cells showed some morphological changes of apoptosis including membrane blebbing (MB), nuclear compaction (NC), cellular shrinkage (CS), and apoptotic bodies (AB) (Figure 2B).

### 3.3 Sapodilla leaf 70% ethanol extract induces G_0_/G_1_ cell cycle arrest in HGT-1 cell line

**FIGURE 3:**
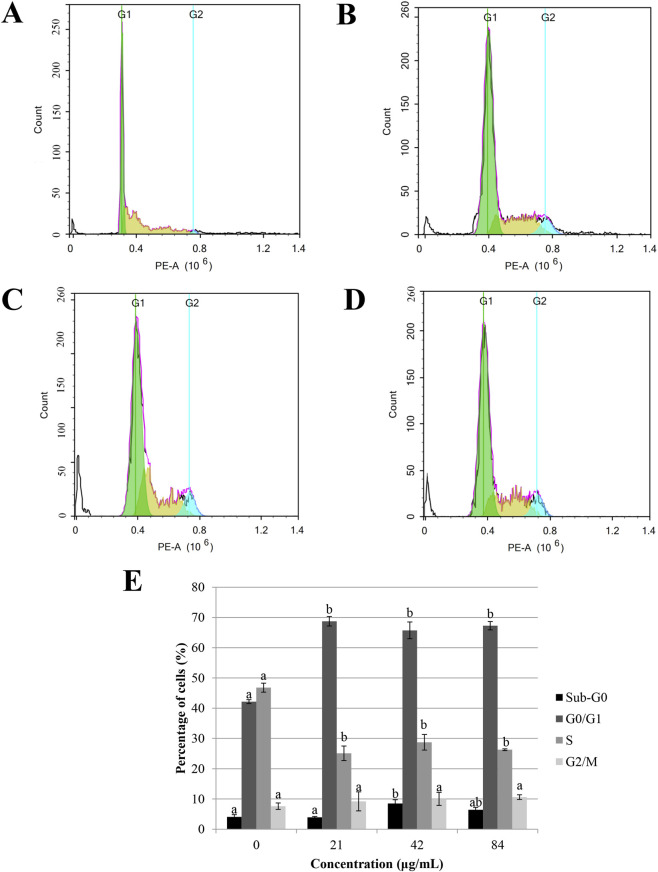
Cell cycle kinetics in **(A)** untreated HGT-1 cells and HGT-1 exposed to sapodilla leaf 70% ethanol extract at concentrations of **(B)** 21 μg/mL, **(C)** 42 μg/mL, and **(D)** 84 μg/mL for 72 h, and assessed using flow cytometry. **(E)** The cell cycle analysis was evaluated using propidium iodide staining and determined by flow cytometry. The data are reported as mean ± SD (n = 3). The data with different superscript letter indicates a significant difference between groups by Tukey’s test (*P* < 0.05).

To determine the cell cycle phase in which the sapodilla leaf 70% ethanol extract possesses growth inhibitory activity, the DNA content of sapodilla leaf 70% ethanol extract was measured using a fluorescence-activated flow cytometer (Figures 3A–D). The previous study has reported that some anticancer agents trigger apoptosis or cell cycle arrest at G_0_/G_1_ and G_2_/M phases and thereby induce apoptosis to kill the cancer cells (Shendge et al., 2021; Tan et al., 2022). As displayed in Figure 3, incubation of 72 h with 42 μg/mL of sapodilla leaf 70% ethanol extract leads to an increase in the sub-G_0_ population. Our findings also showed that sapodilla leaf 70% ethanol extract elevated the number of cells in the G_0_/G_1_ phase followed by a relatively reduced of the S phase (Figure 3E), suggesting that this extract may potentially inhibits gastric cancer cells through cell cycle arrest at G_0_/G_1_ phase.

### 3.4 Sapodilla leaf 70% ethanol extract triggers apoptosis in HGT-1 cells

**FIGURE 4:**
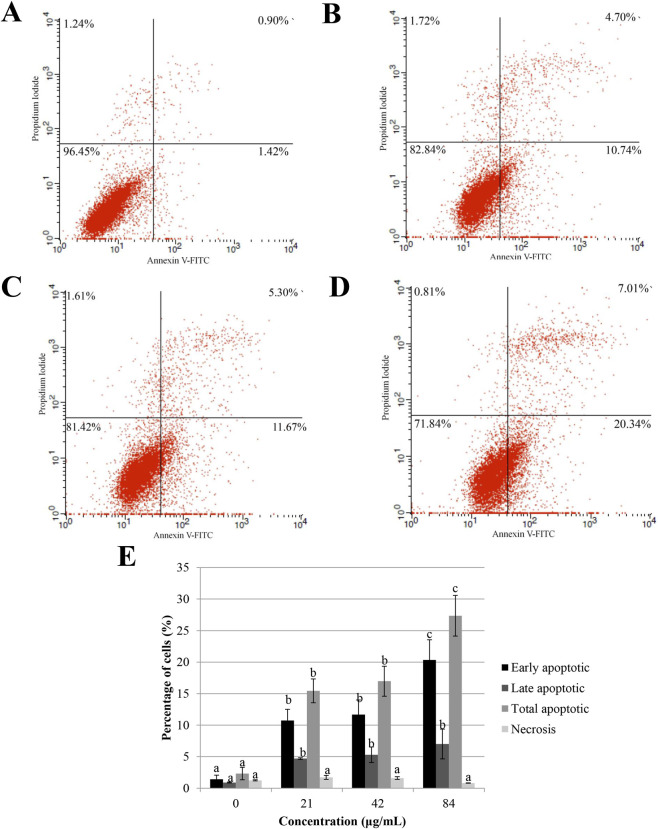
Assessment of sapodilla leaf 70% ethanol extract triggered apoptotic cell death in **(A)** untreated HGT-1 cells and HGT-1 cells exposed to **(B)** 21 μg/mL, **(C)** 42 μg/mL, and **(D)** 84 μg/mL of sapodilla leaf 70% ethanol extract for 72 h. **(E)** Evaluation of apoptotic cell death with sapodilla leaf 70% ethanol extract by flow cytometry using Annexin V-FITC and propidium iodide staining assay. The data are reported as mean ± SD (n = 3). The data with different superscript letter indicates a significant difference between groups by Tukey’s test (*P* < 0.05).

The apoptosis induction by sapodilla leaf 70% ethanol extract in HGT-1 cells was confirmed by Annexin V-FITC study (Figures 4A–D). Our results showed that sapodilla leaf 70% ethanol extract induced dose-dependent apoptosis in HGT-1 cells (Figure 4E). The data showed an upsurge in early apoptosis with the increasing concentration of sapodilla leaf 70% ethanol extract. At zero dose (untreated HGT-1 cells), 1.42% ± 0.66% cells were found in the early apoptotic phase, which was increased to 10.74% ± 1.78%, 11.67% ± 2.32%, and 20.34% ± 3.21% with 21, 42, and 84 μg/mL concentrations, respectively, suggesting that sapodilla leaf 70% ethanol extract kills HGT-1 cells through apoptosis and not via necrosis. Collectively, apoptotic cell percentage increased with concentration, peaking at 84 μg/mL (Figure 4E). The results from the dose-dependent flow cytometry study implied that sapodilla leaf 70% ethanol extract is effective in the apoptosis of HGT-1 cells, suggesting that further elucidations are needed to clarify the molecular pathways regulating apoptosis and cell cycle arrest.

### 3.5 Sapodilla leaf 70% ethanol extract increases bax and decreases Bcl-2 protein levels in HGT-1 cell line

**FIGURE 5:**
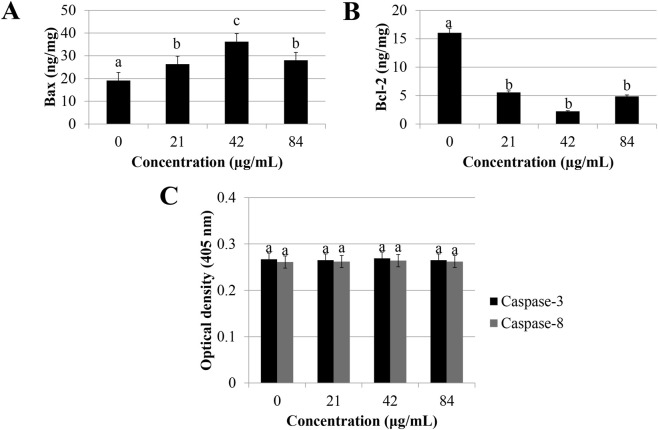
Apoptotic activities of sapodilla leaf 70% ethanol extract on HGT-1 cells after 72 h incubation. HGT-1 cells treated with 21, 42, and 84 μg/mL of sapodilla leaf 70% ethanol extract upregulated apoptotic protein level of **(A)** Bax and downregulated **(B)** Bcl-2, respectively. **(C)** Sapodilla leaf 70% ethanol extract does not activate caspase-3 and -8 activities. The data are reported as mean ± SD (n = 3). The data with different superscript letter indicates a significant difference between groups by Tukey’s test (*P* < 0.05).

Apoptosis is regulated through multiple signaling cascades and involves the activation of various target molecules (Carneiro and El-Deiry, 2020; Yue and López, 2020). Apoptosis can be initiated through mitochondria-independent or mitochondria-dependent pathway (Wang et al., 2020). The intrinsic or mitochondria-mediated signaling is triggered by the family of Bcl-2 proteins and hence controlled the abortion and activation of apoptosis (Qian et al., 2022). To evaluate the molecular pathways of sapodilla leaf 70% ethanol extract in HGT-1 cells, the anti-apoptotic and pro-apoptotic protein expression following sapodilla leaf 70% ethanol extract treatment was assessed (Figure 5). It was shown that incubation with sapodilla leaf 70% ethanol extract for 72 h leads to an elevation of Bax (Figure 5A) and diminished the Bcl-2 protein levels (Figure 5B). Our data suggest that sapodilla leaf 70% ethanol extract triggers apoptosis in HGT-1 cells by downregulating Bcl-2 and upregulating Bax protein levels.

### 3.6 Sapodilla leaf 70% ethanol extract triggers caspase-independent signal transduction pathway

Caspases function as key cysteine proteases in the apoptotic process (Prasad et al., 2022). The results showed that incubation with sapodilla leaf 70% ethanol extract for 72 h was not induced caspase-8 and -3 activities (Figure 5C), implied that sapodilla leaf 70% ethanol extract triggers caspase-independent signal transduction pathway. Although caspases are widely recognized as key executioners of apoptosis, emerging evidence indicates that apoptotic cell death can also proceed via caspase-independent mechanisms, involving other proteolytic enzymes such as cathepsins and alternative proteases (Constantinou et al., 2009). The observed effect in this study is in accordance with the findings demonstrated by Tor et al. (2015), who reported that induction of apoptosis by *Dillenia suffruticosa* ethyl acetate extract was not activated caspase signaling in MCF-7 human adenocarcinoma breast cancer cell line. This observation implies that while caspase activation is likely to be critically involved in apoptosis, it is not the sole determinant, indicating the potential involvement of alternative apoptotic pathways (Blagosklonny, 2000). Further, other significant hallmarks, for example, cell detachment from the substratum and cellular shrinkage have also been implicated in the initiation of caspase-independent apoptotic pathways (Orchel et al., 2021; Leist and Jaattela, 2003).

### 3.7 Sapodilla leaf 70% ethanol extract downregulates NF-κB mRNA expression in HGT-1 cells

**FIGURE 6:**
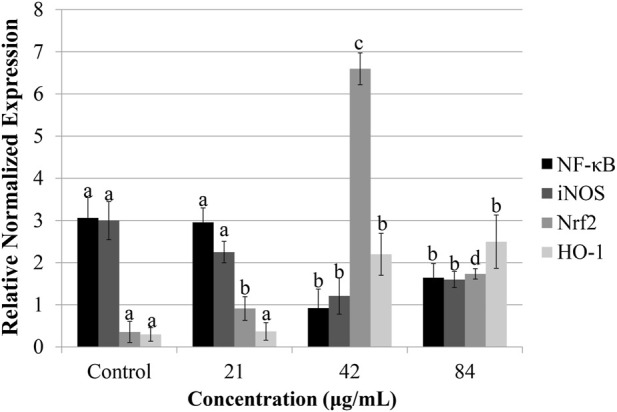
Nuclear factor-kappa B (*NF-κB*), inducible nitric oxide synthase (*iNOS*), nuclear factor E2-related factor 2 (*Nrf2*), and heme oxygenase-1 (*HO-1*) mRNA expression in HGT-1 cells treated with sapodilla leaf 70% ethanol extract for 72 h and accessed by quantitative real-time PCR. The data are reported as mean ± SD (n = 3). The data with different superscript letter indicates a significant difference between groups by Tukey’s test (*P* < 0.05).

NF-κB is a sequence-specific expression associated with inflammatory responses (Chawla et al., 2021). Aberrant stimulation of NF-κB has been implicated in the enhanced expression of genes that facilitate cellular growth and inhibit apoptotic processes (Tan et al., 2016; Deka and Li, 2023). NF-κB is not only modulated the inflammatory process but also acts as a central mediator in tumorigenesis (Mirzaei et al., 2021). We hypothesized that sapodilla leaf 70% ethanol extract downregulates the *NF-κB* expression. Expectedly, untreated HGT-1 cells showed the highest *NF-κB* level than other treated groups (Figure 6). NF-κB expression was significantly reduced in HGT-1 cells treated with 84 and 42 μg/mL sapodilla leaf 70% ethanol extract compared to the untreated control (*P* < 0.05). This observation implied that this treatment can result in the suppression of *NF-κB* mRNA level, with the highest effect observed in 42 μg/mL sapodilla leaf 70% ethanol extract. Some studies demonstrated that tumor cells with activated NF-κB activity were not sensitive to chemotherapeutic agents, whereas suppression of NF-κB expression may promote the chemosensitivity of cancer treatment (Mirzaei et al., 2022; Pan et al., 2016). Accordingly, these findings indicated that sapodilla leaf 70% ethanol extract might be useful in gastric cancer via suppression of NF-κB gene expression. Because active NF-κB can translocate to the nucleus from the cytosol, and subsequently stimulate the *iNOS* expression (Huang et al., 2013), and thus *iNOS* mRNA expression in HGT-1 cells was assessed to evaluate whether this extract could mediate *iNOS* mRNA expression.

### 3.8 Sapodilla leaf 70% ethanol extract downregulates iNOS mRNA expression in HGT-1 cell line

Research evidence has shown that iNOS level is higher in most of the tumor tissue compared to normal tissue (Mintz et al., 2021). iNOS produces nitric oxide and thereby triggers a proinflammatory response to chronic inflammation and leading to cancer progression (Khan et al., 2020). Our study showed that untreated HGT-1 cells had a high *iNOS* mRNA expression. The HGT-1 cells exposed to sapodilla leaf 70% ethanol extract at 42 and 84 μg/mL significantly downregulated *iNOS* mRNA levels compared to the untreated HGT-1 cells (*P* < 0.05) (Figure 6). Downregulation of *iNOS* transcriptional activity in this study was parallel with the data demonstrated by Sheng et al. (1998) and Di Popolo et al. (2000), who showed that high iNOS protein and mRNA expression might contribute to the suppression of apoptosis in colon cancer cells. Another study further revealed that celastrol suppresses the proliferation of colorectal cancer cell line through downregulation of iNOS expression (Gao et al., 2019). Thus, downregulating *iNOS* transcriptional activity may involve in the inhibition of HGT-1 cells, as evidenced by an increase in apoptotic cells.

### 3.9 Sapodilla leaf 70% ethanol extract induces Nrf2 gene expression in HGT-1 cells

The transcriptional factor *Nrf2* can bind with ARE in the promoter of phase II detoxifying enzymes, for instance, HO-1 (Serafini et al., 2020). Thus, we evaluated whether sapodilla leaf 70% ethanol extract could trigger *Nrf2* activation. Our data demonstrated that treatment with sapodilla leaf 70% ethanol extract activates the *Nrf2* gene expression in HGT-1 cell line compared to the control (Figure 6). The finding implied that manipulation of sapodilla leaf 70% ethanol extract in HGT-1 cells may lead to changes in *Nrf2* gene expression, suggesting that this extract is a positive regulator of Nrf2 signaling. Taken together, this observed effect may lead to antioxidant and anti-inflammatory actions. Further, research evidence has revealed that stimulation of the Nrf2 pathway ameliorates oxidative stress in cancer cell proliferation via generation of phase II antioxidant enzymes (Tungalag et al., 2022). Thus, we further assessed the *HO-1* transcriptional activity on sapodilla leaf 70% ethanol extract.

### 3.10 Sapodilla leaf 70% ethanol extract induces Nrf2-Regulated HO-1 transcriptional activity in HGT-1 cells

HO-1 is a key antioxidant enzyme regulated by Nrf2, playing an essential role in modulating intracellular reactive oxygen species (ROS) levels under various stress conditions. HO-1 not only regulates cellular antioxidative defenses but also exhibits anti-inflammatory functions (Campbell et al., 2021). Since HO-1 expression is directly modulated by Nrf2, we further evaluated the effect of sapodilla leaf 70% ethanol extract on *Nrf2*-regulated *HO-1* gene expression in HGT-1 cells. As depicted in Figure 6, the finding revealed that the cells exposed to sapodilla leaf 70% ethanol extract at 42 and 84 μg/mL exhibited upregulation of *HO-1* expression. This finding revealed that treatment of this extract led to an elevation of *HO-1* level, and the maximum effect was noted in the dosage of 84 μg/mL (Figure 6). Collectively, our data suggest that sapodilla leaf 70% ethanol extract prevents inflammatory responses, which could be mediated via stimulation of *Nrf2/HO-1* signaling.

Screening of phytochemical is a procedure used to detect the presence of bioactive constituents in plants. In general, phytochemicals are generally divided into three major groups including phenolic compounds, terpenoids, and alkaloids (Chiocchio et al., 2021). Among the plant chemicals, phenolic compounds play a crucial role in dietary applications due to its protective activity against chronic diseases (Tan et al., 2018c; Vázquez-Ruiz et al., 2023). Research evidence has shown the additive and/or synergistic protective effect of bioactive compounds (Tan et al., 2015; Tan et al., 2018a; Tan et al., 2018b). Phytochemical screening of sapodilla leaf 70% ethanol extract revealed detectable levels of saponins and flavonoids. However, none of the phlobatannins, triterpenoids, and steroids was found in the plant extract. Moreover, we also found that sapodilla leaf 70% ethanol extract contains total flavonoid (0.01 ± 0.003 mg QE/100 g), total phenolic (1.42 ± 0.05 mg GAE/g), phenolic compounds (p-coumaric acid (0.35 ± 0.01 μg/g) and ferulic acid (0.93 ± 0.04 μg/g)), and antioxidant activity as measured using β-carotene bleaching test (48.59% ± 9.52%) and DPPH radical scavenging capacity (0.25 ± 0.02 mg/mL). Collectively, the observed suppression of cell growth and induction of apoptosis may, in part, be attributed to the additive or synergistic interaction between antioxidant activity and bioactive constituents present in the 70% ethanol extract of sapodilla leaf. Although our findings demonstrate concomitant activation of Nrf2/HO-1 and apoptosis, inhibition or knockdown experiments and inclusion of AIF/EndoG are essential to clarify the mechanistic role of this pathway in future studies.

## 4 Conclusion

Our study has shown that sapodilla leaf 70% ethanol extract can suppress the proliferation of HGT-1 cells, activate *HO-1* and *Nrf2*, and inhibit *NF-κB* and *iNOS* mRNA levels. However, in-depth studies should be conducted to define the exact molecular cascades responsible, perhaps in the protein levels, to better evaluate the possible role of this extract on gastric cancer. Direct measurement of ROS levels would provide mechanistic clarity on the oxidative stress driving these responses. Similarly, validation across additional cell lines would strengthen the generalizability of our findings beyond the current model. Assessing caspase-independent markers such as AIF or EndoG could help delineate alternative apoptotic pathways engaged under these conditions. Together, these directions represent important opportunities for future work to expand and deepen understanding of the complex interplay of sapodilla leaf 70% ethanol extract in HGT-1 cells. Because sapodilla leaf 70% ethanol extract is a crude extract, numerous phytochemicals may act differently and thereby many targets might be associated with sapodilla leaf 70% ethanol extract-induced apoptosis in HGT-1 cells. Therefore, our data warrant further in-depth investigations of each bioactive constituent present in the extract for determination of the target involved in anticancer activity against HGT-1 cells. If sapodilla leaf 70% ethanol extract has potential implications against gastric cancer, it will bring local herbs to the forefront of drug discovery. The inhibitory effects of sapodilla leaf 70% ethanol extract against HGT-1 cells may provide some basic evidence for the potential anticancer effects of this plant extract. However, future investigations including *in vivo* models and human trials are essential to confirm the therapeutic potential.

## Data Availability

The original contributions presented in the study are included in the article/supplementary material, further inquiries can be directed to the corresponding author.
